# Validation of a German Version of the Grief Cognitions Questionnaire and Establishment of a Short Form

**DOI:** 10.3389/fpsyg.2020.620987

**Published:** 2021-01-18

**Authors:** Bettina K. Doering, Paul A. Boelen, Maarten C. Eisma, Antonia Barke

**Affiliations:** ^1^Clinical and Biological Psychology, Catholic University Eichstaett-Ingolstadt, Eichstaett, Germany; ^2^Clinical Psychology, Utrecht University, Utrecht, Netherlands; ^3^ARQ National Psychotrauma Centre, Diemen, Netherlands; ^4^Department of Clinical Psychology and Experimental Psychopathology, University of Groningen, Groningen, Netherlands

**Keywords:** grief, bereavement, cognition, validation study, questionnaire

## Abstract

**Background:**

Whereas the majority of bereaved persons recover from their grief without professional assistance, a minority develops pathological grief reactions. Etiological models postulate that dysfunctional cognitions may perpetuate such reactions. The Grief Cognitions Questionnaire (GCQ) assesses thoughts after bereavement in nine interrelated domains. A short form (GCQ-SF) with four domains is often used. However, an evaluation of the psychometric properties of the GCQ-SF and its utility compared to the GCQ is lacking and these instruments have not been validated in German.

**Method:**

German bereaved persons (time since loss 35.3 ± 34.6 months) responded to an online survey containing the GCQ, measures of grief severity, grief rumination, symptoms of depression and anxiety, and optimism and pessimism. 585 participants (18–78 years, 88% women) were included. Item analyses and confirmatory factor analyses were conducted. Correlations between the GCQ and GCQ-SF and grief rumination, optimism and pessimism assessed construct validity. Criterion-related validity was assessed by comparing whether the correlation of the GCQ (and the GCQ-SF) with grief severity was higher than with anxious and depressive symptoms. Logistic regression and receiver-operator characteristics (ROC) compared the questionnaires on their ability to predict probable prolonged grief ‘caseness’ (ICG ≥ 25, time since loss ≥6 months).

**Results:**

Internal consistencies for both questionnaires were identical and excellent (α = 0.96). Confirmatory factor analyses obtained a satisfactory fit for models with nine and four correlated subscales and respective higher-order factor models. The GCQ and the GCQ-SF correlated higher with grief severity than with other measures of psychopathology. The logistic regression showed a significant association between the GCQ-SF and prolonged grief ‘caseness’. Of the remaining subscales of the GCQ, only one subscale (‘Others’) contributed to the prediction. The ROC analyses showed nearly identical areas under the curve.

**Conclusion:**

The translated GCQ and GCQ-SF demonstrated very good psychometric properties. The correlations with grief severity highlight the questionnaires’ clinical relevance. The questionnaires possessed identical diagnostic specificity and sensitivity. Whenever a timesaving assessment of the most typical grief-specific cognitions is important, the GCQ-SF represents an alternative to the GCQ. The original GCQ may still be superior when a more detailed description of a bereaved person’s cognitions is desirable.

## Introduction

While losing a loved one can be a painful and distressing life event, most people adjust to it in due time. For a minority of the bereaved, however, grief does not abate and becomes what the ICD-11 terms Prolonged Grief Disorder (PGD) (World Health Organization [WHO], 2020) and the DSM 5 labels Persistent Complex Bereavement Disorder (PCBD) (American Psychiatric Association [APA], 2013). The classification systems differ with respect to certain characteristics of the condition: most importantly, while the ICD-11 only requires a time criterion of 6 months for establishing a PGD diagnosis, the DSM-5 sets a time criterion of 12 months for PCBD. Additionally, accessory symptoms of the respective disorders vary [cf. (Boelen et al., 2018) for an overview and empirical investigation and (Boelen et al., 2020) for a commentary on the recently proposed changes to PCBD]. Both classification systems agree, however, that the disorder is characterized by intense yearning for the deceased person, pervasive cognitive preoccupation with the deceased and emotional distress due to the separation. This article will refer to both conditions as ‘prolonged grief’ to indicate pathological grief processes in general. Prolonged grief is associated with clinically significant impairment and negative health outcomes (Prigerson et al., 1997; Maccallum and Bryant, 2019). Its estimated prevalence among bereaved persons in general ranges from 6.7% (Kersting et al., 2011) to 9.8% (Lundorff et al., 2017). When considering only persons bereaved by violent losses, estimates have been as high as 49% (Djelantik et al., 2020).

Various theoretical conceptions have been put forward to explain how normal grief can turn into prolonged grief. Among them is the cognitive behavioral model by Boelen et al. (2006b), which has received considerable empirical support. This model proposes that individual risk factors (e.g., the relationship to the deceased, loss characteristics) influence grief symptoms through three mediating and interacting core processes, which are central to the development and the maintenance of the disorder. These three processes are (a) insufficient integration of the loss into the autobiographical knowledge base; (b) negative global beliefs and misinterpretations of grief reactions; and (c) anxious and depressive avoidance strategies. Thus, negative cognitions play an important role in this model, but also in other etiological conceptualizations (Parkes, 1988; Schwartzberg and Janoff-Bulman, 1991; Rando, 1993).

In the cognitive behavioral model, negative cognitions can exert their influence through different pathways (Boelen et al., 2006b): They may lead directly to aversive emotional states consistent with prolonged grief such as emotional pain, yearning or sadness. Negative cognitions may also encourage situational and cognitive avoidance strategies blocking emotional processing. Finally, negative cognitions may prevent the loss from becoming integrated in the autobiographical memory, e.g., because elaborating the loss and its implications triggers painful negative thoughts. Negative cognitions may thus contribute to the development and maintenance of prolonged grief symptoms.

Importantly, and in contrast to the aforementioned potential risk factors (loss characteristics, etc.), negative cognitions are modifiable. They can be the target of prevention and treatment of prolonged grief (Doering and Eisma, 2016). In order to be able to target cognitions for modification, it is of high relevance to assess and identify grief-related negative cognitions. To this end, the ‘Grief Cognitions Questionnaire’ (GCQ) (Boelen and Lensvelt-Mulders, 2005) was developed as a measure of negative thought content after bereavement.

The GCQ captures nine grief-specific negative belief themes. Among them are negative cognitions about the self (‘Since he/she is dead, I am of no importance to anybody anymore’), the world (‘His/her death has taught me that the world is unjust’), one’s life (‘My life is meaningless since he/she died’), and the future (‘Since he/she is no longer here, I have a negative view on the future’). On the one hand, these beliefs may develop after being confronted with the death of a loved one because the loss challenges and changes pre-existing more positive beliefs (Schwartzberg and Janoff-Bulman, 1991). On the other hand, the loss may also strengthen already existing negative beliefs (Thimm and Holland, 2017). Another belief theme concerns cognitions related to self-blame (‘I will never be able to forgive myself for the things I did wrong in the relationship with him/her’) that may hinder the resolution of grief (van der Houwen et al., 2010; Stroebe et al., 2014). These include self-reproach focused on having caused the death, not having prevented it or for having made non-redressable mistakes in the relationship with the deceased. A further theme encompasses negative evaluations of the available social support after the loss (‘Many people have let me down since his/her death’). Negative evaluations of the social environment’s reactions are associated with poorer health outcomes in bereavement (van der Houwen et al., 2010). The remaining themes consider cognitions about one’s own grief reactions. Such cognitions may complicate the grieving process (Malkinson, 1996). Some mourners may interpret their grief reactions as dangerous (‘Once I start crying, I will lose control’), which may promote grief-related experiential avoidance (Boelen et al., 2010). Others may be concerned about the appropriateness of their grief reactions (‘I don’t mourn the way I should do’). Finally, cognitions may reflect a perceived necessity to cherish one’s grief as a means to maintaining a relationship with the deceased (‘As long as I mourn, I do not really have to let him/her go’). In some cases, such beliefs may also hinder adjustment to a reality without the loved person (Stroebe and Schut, 2005). The nine subscales of the GCQ reflect these grief-specific negative belief themes.

The GCQ was established initially in a sample of bereaved persons who experienced the death of a first-degree relative (Boelen et al., 2003). Various studies examined its psychometric properties. Robust evidence speaks for the GCQ’s reliability (Cronbach’s α = 0.96 for the total scale and 0.81 ≤ Cronbach’s α ≤ 0.95 for the subscales) and temporal stability (*r*_test–retest_ = 0.94 and 0.85 after three and four-week retest-intervals, respectively) (Boelen and Lensvelt-Mulders, 2005). Its conceptualized factorial structure of nine interrelated factors (Boelen et al., 2003) has been confirmed (Boelen and Lensvelt-Mulders, 2005).

Concerning its validity, the GCQ total score was positively associated with pessimism and behavioral avoidance of bereavement cues, while it correlated negatively with measures of positive thinking and optimism (Boelen and Lensvelt-Mulders, 2005; Cesur and Durak-Batıgün, 2021). Since the GCQ was designed as a measure of negative (bereavement-related) thinking, the positive association between the GCQ and pessimism and its negative association with optimism speak for its convergent and discriminant validity. The relationship between negative grief-specific cognitions and grief rumination (Boelen and Lensvelt-Mulders, 2005) also underlines the convergent validity of the GCQ. While the GCQ assesses negative cognitions and their endorsement by bereaved participants, grief rumination assesses the frequency with which participants engage in the process of repetitive and recurrent thinking about causes and consequences of the loss and loss-related emotions (Eisma et al., 2014). Thus, the cognitions specified in the GCQ may be viewed as part of the cognitive ‘content’ that is repetitively processed and activated in grief rumination. In accordance with the Response Style Theory (Nolen-Hoeksema et al., 2008), rumination contributes to bereavement-related distress by increasing the accessibility of negative cognitions (Eisma and Stroebe, 2017).

Regarding its criterion validity, the GCQ classified correctly (87.8%) probable ‘caseness’ for prolonged grief (Boelen and Lensvelt-Mulders, 2005). Further studies demonstrated that this association remained significant even when controlling for depressive symptoms (Liu et al., 2019). The GCQ is positively associated with grief severity: When considering grief severity as a continuous variable, all GCQ subscales explained a significant amount of variance over and above loss-related and sociodemographic variables (Boelen et al., 2003), and symptoms of depression and anxiety (Boelen and Lensvelt-Mulders, 2005; Cesur and Durak-Batıgün, 2021). Participants who were identified by self-report as candidates for prolonged grief demonstrated higher scores for the total scale and all subscales even when controlling for loss-related characteristics (Boelen et al., 2003; Boelen and Lensvelt-Mulders, 2005). Thus, research has demonstrated a close and specific association of the GCQ with prolonged grief over and above the contribution of loss-related factors, sociodemographic variables and indicators of other psychopathology.

As the whole scale is quite long (38 items), subsequent research often used combinations of GCQ subscales instead of the full GCQ. The use of a limited number of subscales makes the questionnaire more time-efficient. This is of special importance in grief research, since bereaved individuals may be highly distressed and long questionnaires may add to the response burden in surveys (Rolstad et al., 2011). Since the four subscales ‘Life’, ‘Self’, ‘Future’, and ‘Threatening Interpretations of Grief’ [sometimes also termed ‘Catastrophic Misinterpretations’ (Boelen and Lenferink, 2020)] were concurrently and prospectively most strongly associated with poorer adjustment to bereavement (Boelen et al., 2003, 2006a; Boelen and Lensvelt-Mulders, 2005), their combination is the most frequently used GCQ short form (GCQ-SF). In spite of its frequent use in research, a thorough psychometric analysis of this short form has not yet been undertaken.

Several studies have investigated the GCQ-SF and found evidence for its association with grief severity (Boelen and Klugkist, 2011; Shi et al., 2019; Boelen and Lenferink, 2020). In a sample of very recently bereaved individuals, a latent class analysis demonstrated significant associations between membership of grief symptom profiles and negative cognitions as measured by the GCQ-SF (Boelen and Lenferink, 2020). Especially the subscale ‘Threatening Interpretations of Grief’ was a significant predictor for overall symptom burden, thus underscoring the importance of negative cognitions for bereavement outcome. In a sample of bereaved persons who were surveyed at three time points (less than five months after the loss, and six and 15 months later, respectively), the four GCQ-SF subscales were related to grief severity, both concurrently and longitudinally, even after controlling for relevant sociodemographic and loss-related variables (Boelen et al., 2006a). When baseline grief severity was taken into account, all GCQ-SF subscales predicted grief severity at the second assessment, and ‘Life’ and ‘Future’ even at the third. Notably, the GCQ-SF has not only been used in observational studies but has also served as secondary outcome in a study investigating cognitive behavioral therapy for prolonged grief (Boelen et al., 2011). In this study, a reduction in the subscale scores after grief-specific psychotherapy was associated with better treatment outcome (i.e., greater reduction in grief severity), both at post-treatment and at follow-up.

This considerable body of evidence suggests that the GCQ is a reliable, change-sensitive, and valid instrument to assess negative cognitions after bereavement. Instruments such as the GCQ are highly relevant: the recognition of prolonged grief as a disorder in the international classification systems ICD-11 and DSM 5 underlines the need to assess etiological factors that contribute to this disorder, such as negative grief-specific cognitions. Additionally, there is a need for validated translations of these questionnaires: prolonged grief and grief in general must be considered with special regard to cultural differences in the duration of symptoms and the expression of grief (Killikelly and Maercker, 2017). To further international, cross-cultural empirical research, validated translations become ever more important. Concerning the GCQ, which is available in Dutch and English, subscales have been translated to French (Kokou-Kpolou et al., 2018); formal validation studies have been conducted for a Turkish version (Cesur and Durak-Batıgün, 2021) a Jordanian version (Basim and Noor, 2020), and a Chinese version (Yu et al., 2014). A German version of the GCQ, however, is lacking, as is a validation study for the GCQ short form.

The first aim of the present study was therefore to establish and validate a German version of the GCQ to further its international availability. We expected the German GCQ to demonstrate psychometric properties comparable to the original version. The second aim was to investigate the psychometric properties and factorial structure of the GCQ short form (comprising the subscales ‘Life’, ‘Self’, ‘Future’, and ‘Threatening Interpretations of Grief’). We predicted that the GCQ-SF would show psychometric properties mostly comparable to the GCQ; a slightly lower reliability could be expected due to the shortening of the scale (as Cronbach’s alpha increases with the number of items). Concerning the factorial structure, we expected an acceptable model fit for a second-order four-factor model representing the four included subscales on the first level and a second-level general factor. In an exploratory analysis, we investigated the associations between the GCQ and the GCQ-SF with sociodemographic variables, i.e., age and gender. Lastly, we aimed to assess the original GCQ and the GCQ-SF with regard to their construct and criterion-related validity. With regard to construct validity, we predicted that the GCQ and the GCQ-SF scores would be associated positively with grief rumination and pessimism, and negatively with optimism. Regarding criterion-related validity, we made three predictions: First, that the GCQ and the GCQ-SF would be associated with grief severity. Second, that the GCQ and GCQ-SF would be more strongly associated with grief severity than with symptoms of anxiety or depression. Third, that the GCQ and the GCQ-SF would be strongly related to probable ‘caseness’ of prolonged grief, i.e., belonging to a high-risk group for prolonged grief.

## Materials and Methods

### Procedures

Ethical approval (2016-39k) was obtained from the Ethics Committee of the Department of Psychology from the Philipps-University Marburg (Germany). Recruitment lasted from April to December 2017. Invitations for the study, including a link to an online survey platform, were posted on grief-related websites (e.g., peer support websites) and sent via mailing lists of the university (staff and students). The survey platform provided information about e.g., study aims, confidentiality and study eligibility criteria. Inclusion criteria were age ≥18, having lost a loved one within the last 10 years and being a German native speaker. Exclusion criteria were suicidal ideation or anticipating feeling too distressed by loss-related questions. Criteria for inclusion and exclusion were assessed by self-report. Median time to complete the questionnaire was 17 min (ranging from 7 min to 46 min). Participants received no compensation for completing the survey.

### Measures

#### Demographic and Loss-Related Variables

In addition to sociodemographic data (age, gender, native language, educational level) participants also provided loss-related data. First, participants were asked which losses they had ever experienced (i.e., spouse/partner, child, sibling, parent, grand-parent, other). Next, they indicated which loss was still most distressing to them. For this loss, additional data were collected: time since loss (indicated by date of death and three categories: less than 6 months, 6-12 months, more than a year); relationship to the deceased (i.e., spouse/partner, child, sibling, parent, grand-parent, other); cause of death (natural, accident, suicide, homicide, other); and how the participants had experienced the death (expected, unexpected, both/neither).

#### Grief Cognitions Questionnaire

Two independent psychologists [BD and LB (cf. acknowledgments)] translated the English version of the Grief Cognitions Questionnaire into German. The versions were reviewed and compared for differences indicating a different understanding of the original items. Both translations were very close and were subsequently merged by consensus into one German version. This consensus version was back-translated (AB) following the guidelines by Beaton et al. (2000). The back-translated questionnaire was then discussed with the original author (PB) for semantic equivalence. The final German version is provided as Supplementary Material 1. The GCQ is a 38-item questionnaire that measures grief-related negative cognitions (Boelen et al., 2003; Boelen and Lensvelt-Mulders, 2005). Participants are presented with 38 cognitions as statements and indicate the extent to which they agree with the respective statement on a 6-point Likert scale (0 = disagree strongly, 5 = agree strongly). The GCQ comprises nine inter-correlated subscales (Boelen and Lensvelt-Mulders, 2005). The subscale ‘Self’ encompasses global negative beliefs about the self since the loss (6 items). ‘World’ describes a negative view of the world since the loss (4 items). ‘Future’ comprises negative views on the future without the deceased (5 items). ‘Life’ describes negative views concerning the meaning of one’s life since the death (4 items). ‘Self-blame’ encompasses cognitions of not having prevented the death or regrets about one’s role in the relationship with the deceased (5 items). ‘Others’ encompasses negative evaluations of the available social support after the loss (3 items). ‘Appropriateness of Grief’ describes negative evaluations of one’s own grief reactions (4 items). ‘Cherish Grief’ reflects beliefs about the importance of cherishing the pain of the loss (3 items). ‘Threatening Interpretations of Grief’ contains catastrophic misinterpretations of in themselves harmless symptoms of grief (4 items). A total score is calculated by summing all items, and subscale scores by summing the respective items for each subscale. In previous research, the reliability of the total scale was excellent (Cronbach’s α = 0.96), with high to excellent reliability for the subscales (0.81 ≤ Cronbach’s α ≤ 0.95) (Boelen and Lensvelt-Mulders, 2005). As noted, several studies have used an abbreviated version of the GCQ, i.e., the GCQ-SF, containing only the subscales ‘Self’, ‘Life’, ‘Future’, and ‘Threatening Interpretations of Grief’(Boelen et al., 2006a, 2011).

#### Inventory of Complicated Grief

The Inventory of Complicated Grief (ICG) (Prigerson et al., 1995) was used in its German version (ICG-D) (Lumbeck et al., 2013). Its 19 items encompass emotional, cognitive and behavioral states relevant to prolonged grief (e.g., ‘I feel myself longing for the person who died.’). Participants are asked to rate the occurrence of each state on a 5-point scale (0 = never; 4 = always). A total score assesses the severity of grief symptoms by summing of all items. The ICG-D has demonstrated excellent internal consistency (Cronbach’s α = 0.94) and good validity (Lumbeck et al., 2013). In the present sample, Cronbach’s α was α = 0.94. Prigerson and colleagues (Boelen et al., 2011) have established a cut-off (≥25) indicating more disabling states of grief. This cut-off has been used previously to identify probable ‘cases’ of prolonged grief (Kristensen et al., 2010; Newson et al., 2011).

#### Utrecht Grief Rumination Scale

The Utrecht Grief Rumination Scale (UGRS) (Eisma et al., 2014) was used in its German version (UGRS-D) (Doering et al., 2018). Participants rate the frequency with which they have engaged in repetitive thoughts about the loss in the past month on a 5-point scale (1 = never; 5 = very often). Its 15 items form five subscales (three items each), which focus on different themes of rumination about causes and consequences of the loss: (1) personal emotional reactions to the loss (e.g., ‘How often in the past month did you try to analyze your feelings about this loss precisely?’), (2) injustice of the death (e.g., ‘How often in the past month did you wonder why this had to happen to you and not to someone else?’), (3) counterfactual thoughts about the circumstances of the death (e.g., ‘How often in the past month did you analyze if you could have prevented the death?’) (4) meaning and consequences of the loss (e.g., ‘How often in the past month did you analyze what the personal meaning of the loss is for you?’), and (5) the reactions of others to the loss (e.g., ‘How often in the past month did you think about how you would like others to react to your loss?’). A total score of grief rumination is calculated by summing of all items; subscale scores can be obtained by summing the subscale items. The internal consistency of the UGRS-D is good (Doering et al., 2018); in the present sample, Cronbach’s α was α = 0.92.

#### Hospital Anxiety and Depression Scale

The Hospital Anxiety and Depression Scale [HADS (Zigmond and Snaith, 1983)] was used in its German version [HADS-D (Herrmann-Lingen et al., 2011)]. Its two subscales, each consisting of seven items, assess symptoms of anxiety (example item: ‘I feel tense or wound up’) and depression (inverted example item: ‘I feel cheerful’) with regard to the past week. Symptoms are evaluated on a 4-point scale by asking for the frequency of occurrence, intensity of a symptom or associated changes in behavior. Subscale scores can be obtained by summing the respective items, with higher subscale scores indicating higher anxiety and depression, respectively. The subscales have good reliability (anxiety: Cronbach’s α = 0.80; depression: Cronbach’s α = 0.81) (Herrmann-Lingen et al., 2011) and validity. In the present sample, Cronbach’s α was α = 0.92 for depression and α = 0.85 for anxiety.

#### Life Orientation Test-Revised

The Life Orientation Test-Revised (LOT-R) (Scheier et al., 1994) was used in its German version (Glaesmer et al., 2008). It contains ten items, with three items assessing dispositional optimism (e.g., ‘In uncertain times, I always expect the best’), three items assessing dispositional pessimism (e.g., ‘If something can go wrong for me, it will’), and four filler items. Items are rated on a 5-point Likert scale (0 = strongly disagree; 4 = strongly agree). Scores for the two subscales are obtained by summing the respective items with higher scores indicating higher optimism and pessimism, respectively. Its internal consistency is acceptable (Glaesmer et al., 2012). In the present sample, Cronbach’s α for optimism was α = 0.79, for pessimism α = 0.74.

### Participants

A total of 1,121 participants gave informed consent; 864 provided at least demographic data so that they could be assessed with regard to study eligibility. Of these 864 participants, 26 were not German native speakers and seven participants were younger than 18 years, and were thus excluded. Of the 831 eligible participants (100%), 587 completed the survey. Two participants were excluded due to answer patterns (i.e., ‘straightlining’, SD = 0 in all questionnaires) so that the final sample consisted of 585 participants, resulting in a completer rate of 70.4%.

The majority of the sample was female (88.4%). Mean age was 40.2 years (±SD 13.2; range 18–78 years). The majority of the participants reported a higher educational level (i.e., graduate from high school, college or university or advanced technical professional; 66.6%). Participants indicated having experienced the following losses (multiple answers possible): spouse/partner (25.3%), child (15.7%), parent (40.9%), sibling (6.8%), grandparent (21.9%) and another loved one (e.g., friend, 13.3%). Participants were asked to indicate the loss that was still most distressing to them, and report its loss-related characteristics. The participants indicated the following relationships for the most distressing loss: spouse/partner (24.6%), child (14.5%), parent (33.7%), sibling (5.1%), grandparent (13.7%) and other loved one (8.4%). The cause of death was predominantly from a natural cause (68.0%) with the remaining causes of death being accidents (7.2%), suicides (9.1%), homicide (0.9%) or other causes (12.3%). For most participants, the death had been unexpected (56.9%), while 24.1% reported having expected the death and 19.0% described the death as either both expected and unexpected or neither expected nor unexpected. Mean time since loss was 35.3 ± 34.6 months (range 0–131 months, MD = 22.0 months).

### Statistical Analyses

Since the survey set the GCQ items as mandatory, no missing data were observed in the GCQ. Answers to other items were optional. Single missing items in other questionnaires (three single values) were replaced according to the respective questionnaire’s instructions (i.e., replacement of single missing items by mean of the scale/subscale). To investigate the psychometric properties of the GCQ, standard item analyses were calculated: mean item scores and standard deviations, item difficulties, item-total correlations with the item itself excluded from the total score, and estimations of internal consistency when the item was omitted.

The factorial structure of the GCQ and the GCQ-SF was investigated by confirmatory factor analyses (CFA) with maximum likelihood estimation. Of the four models proposed for the GCQ, those two models were tested that had commanded the best empirical support (Boelen and Lensvelt-Mulders, 2005). Model 1 hypothesizes a nine-factor model with correlated factors, which represent the nine GCQ subscales. Model 2 replaces the correlations by a general factor (second-order nine-factor model). For the abbreviated GCQ-SF, we tested analogous models: Model 1-SF stipulated four correlated factors representing the four included subscales. Model 2-SF added to this a general factor (second-order four-factor model). Since the GCQ scores did not meet the assumption of a normal distribution as evident after an inspection of skewness and kurtosis, we performed a log-transformation of the GCQ scores prior to conducting the CFA. To assess goodness of fit, we inspected the χ^2^ test, the root mean square error of approximation (RMSEA) and the standardized root mean squared residual (SRMR). The following are viewed as cut-off values indicating a good fit: χ/df ratio of ≤2 or 3, RMSEA < 0.06 to 0.08 with confidence interval, SRMR < 0.08; and for the Tucker-Lewis -Index (TLI) and the comparative fit index (CFI) ≥ 0.95 (Schreiber et al., 2006). To compare the respective models, we inspected the Akaike Information Criterion (AIC); and the Bayesian Information Criterion (BIC); while their absolute values are not informative, smaller AIC and BIC scores indicate a better model fit when comparing different models.

In exploratory analyses, we investigated the associations of sociodemographic variables, i.e., age and gender with the GCQ and the GCQ-SF. For age, we calculated correlations between age and the total scores of both questionnaires. To investigate the influence of gender, we conducted independent sample *t*-tests to compare men and women with regard to their scores in the GCQ and the GCQ-SF. If Levene’s test indicated that variances were unequal, the Welch test is reported (and the degrees of freedom were adjusted accordingly). Where appropriate, Cohen’s *d* is reported as a measure of effect size.

To investigate further facets of validity, we calculated correlations of the GCQ with grief severity (ICG-D), grief rumination (UGRS-D), optimism and pessimism (LOT-R) and anxiety and depression (HADS-D). To account for possible alpha error inflation due to multiple comparisons, significance levels were Bonferroni-corrected. Construct validity was assessed by inspecting zero-order correlations between the GCQ and grief rumination, pessimism, and optimism. With regard to criterion-related validity, zero-order correlations between the GCQ and grief severity (ICG-D) were calculated. Z-tests compared the zero-order correlations of the GCQ and the ICG-D to correlations of the GCQ with other measures of psychopathology (anxiety and depression). All these analyses were conducted analogously with the GCQ-SF. Additionally, a logistic regression and receiver operator characteristic (ROC) analysis compared the criterion validity of the GCQ and the GCQ-SF. As a binary criterion, the probable prolonged grief ‘caseness’ was operationalized using the cut-off of the ICG (ICG ≥ 25), which has been established in previous research (Kristensen et al., 2010; Newson et al., 2011). Only participants who fulfilled the ICD-11 time criterion (time since loss ≥6 months) were included in this analysis. A block-wise logistic regression (Method: forward selection; Wald) with the criterion group membership was conducted with the GCQ-SF as a first block (model 1) and the remaining five GCQ scales as a second block (model 2). The models were compared concerning the goodness of fit (log-likelihood statistic) and the explained variance (Nagelkerke’s R^2^). Individual predictors were assessed using the Wald statistic and odds ratios. In order to investigate the discriminatory power of the GCQ to predict probable prolonged grief ‘caseness’, ROC’s of the GCQ and the GCQ-SF were calculated. The combined sensitivity and specificity as expressed by the area under the curve (AUC) is reported. Higher values of the GCQ are taken as indicative of probable prolonged grief ‘caseness’.

The data analysis was carried out with IBM SPSS statistics 24; for the confirmatory factor analysis, the SPSS AMOS version 21.0.0 was used (IBM, Meadville, United States). Unless otherwise stated, the α-level was set to *p* = 0.05.

## Results

### Item Analyses

For the GCQ, the internal consistency of the total scale was Cronbach’s α = 0.96. Removing any item from the scale would not have improved its internal consistency (standardized alpha for the subscales if the item was removed was 0.96 for all items). Consistency coefficients for the subscales were: Self α = 0.88; World α = 0.87; Life α = 0.94; Future α = 0.91; Self-Blame α = 0.85; Others α = 0.84; Appropriateness of Grief Reactions α = .85; Cherish Grief α = 0.76; Threatening Interpretations of Grief Reactions α = 0.88. Table 1 presents means and standard deviations for each item. Mean item difficulty was *p*_*i*_ = 0.27 with a range from *p*_*i*_ = 0.12 (item 9) to *p*_*i*_ = 0.43 (items 14, 18, 19). The mean inter-item correlation was *r*_*itc*_ = .40 with item-whole correlations ranging from *r*_*itc*_ = 0.38 (item 6) to *r*_*itc*_ = 0.79 (item 35).

**TABLE 1:** Item means, standard deviations, skewness, kurtosis, item difficulties, and item-whole correlations with the subscales (*n* = 585).

Item	M	SD	Skew	Kurt	Difficulty	Item-whole correlation
1† Since he/she is dead, I think I am worthless.	1.05	1.40	1.24	0.56	0.21	0.74
2 I am partially responsible for his/her death.	0.91	1.38	1.46	1.11	0.18	0.46
3 Since he/she died, I realize that the world is a bad place.	1.13	1.34	1.14	0.44	0.23	0.65
4 The people around me should give me more support.	1.76	1.65	0.62	–0.84	0.35	0.57
5† I don’t expect that I will feel better in the future.	1.76	1.70	0.62	–0.88	0.35	0.67
6 I have to mourn otherwise I will forget him/her.	1.33	1.56	1.01	–0.14	0.27	0.38
7† I see myself as a weak person since he/she passed away.	1.39	1.64	0.94	–0.36	0.28	0.72
8† If I let go of my emotions, I will go crazy.	1.70	1.77	0.67	–0.95	0.34	0.66
9† I am ashamed of myself, since he/she died.	0.58	1.12	2.17	4.26	0.12	0.48
10 His/her death has made me realize that we live in an awful world.	1.29	1.55	1.07	0.01	0.26	0.65
11 My grief reactions are abnormal.	0.86	1.31	1.55	1.41	0.17	0.46
12† Life has got nothing to offer me anymore.	1.00	1.47	1.44	0.95	0.20	0.72
13† I don’t have confidence in the future.	1.21	1.60	1.16	0.07	0.24	0.74
14 As long as I mourn I maintain the bond with him/her.	2.16	1.74	0.24	–1.21	0.43	0.53
15† My life is useless since he/she died.	1.05	1.51	1.42	0.96	0.21	0.77
16 I don’t mourn the way I should do.	1.21	1.51	1.08	–0.01	0.24	0.39
17 I should have prevented his/her death	1.46	1.80	0.85	–0.77	0.29	0.51
18 Many people have let me down after his/her death	2.17	1.90	0.26	–1.42	0.43	0.74
19 His/her death has taught me that the world is unjust.	2.16	1.91	0.26	–1.44	0.43	0.52
20† My life is meaningless since he/she died	1.15	1.57	1.27	0.40	0.23	0.64
21† My wishes for the future will never be fulfilled.	1.67	1.57	0.67	–0.91	0.33	0.76
22† Since he/she is dead, I feel less worthy.	1.18	1.55	1.18	0.22	0.24	0.74
23† If I fully realized what his/her death means, I would go crazy.	1.61	1.77	0.79	–0.79	0.32	0.77
24 If I had done things differently, he/she would still be alive.	1.24	1.66	1.05	–0.28	0.25	0.72
25† Ever since he/she died, I think negatively about myself.	1.14	1.50	1.19	0.27	0.23	0.43
26 I do not react to this loss normally.	1.01	1.40	1.39	1.02	0.20	0.74
27† In the future I will never be really happy anymore.	1.47	1.70	0.88	–0.57	0.29	0.50
28 As long as I mourn I do not really have to let him/her go.	1.51	1.68	0.85	–0.57	0.30	0.75
29 People around me should show much more interest in me.	1.55	1.68	0.79	–0.66	0.31	0.62
30 I will never be able to forgive myself for the things I did wrong in the relationship with him/her.	1.60	1.73	0.75	–0.80	0.32	0.50
31 There is something wrong with my feelings.	0.92	1.40	1.56	1.43	0.18	0.57
32† My life has no purpose anymore, since he/she died.	1.00	1.49	1.44	0.96	0.20	0.53
33 I blame myself for not having cared better for him/her.	2.07	1.73	0.32	–1.31	0.41	0.75
34 His/her death has taught me that the world is a worthless place.	0.96	1.40	1.47	1.17	0.19	0.48
35† Since he/she is no longer here, I have a negative view on the future.	1.43	1.65	0.91	–0.43	0.29	0.79
36† If I allow my feelings to come, I will lose control.	1.52	1.74	0.86	–0.63	0.30	0.73
37† Since he/she is dead, I am of no importance to anybody anymore.	0.81	1.34	1.77	2.27	0.16	0.65
38† Once I start crying, I will lose control.	1.34	1.64	1.02	–0.26	0.27	0.64

*† Items also included in the GCQ-SF. KURT: kurtosis. The standardized alpha for the subscales if the item was removed was.96 for all items.*

For the GCQ-SF, the internal consistency of the total score was α = 0.96. The internal consistency would not improve by omitting any item. The mean item difficulty was *p*_*i*_ = 0.25 with a range from *p*_*i*_ = 0.12 (item 9) to *p*_*i*_ = 0.35 (item 5). The mean inter-item correlation was *r*_*itc*_ = 0.56 with item-whole correlations ranging from *r*_*itc*_ = 0.43 (item 9) to *r*_*itc*_ = 0.84 (items 15, 35).

### Confirmatory Factor Analysis

**FIGURE 1:**
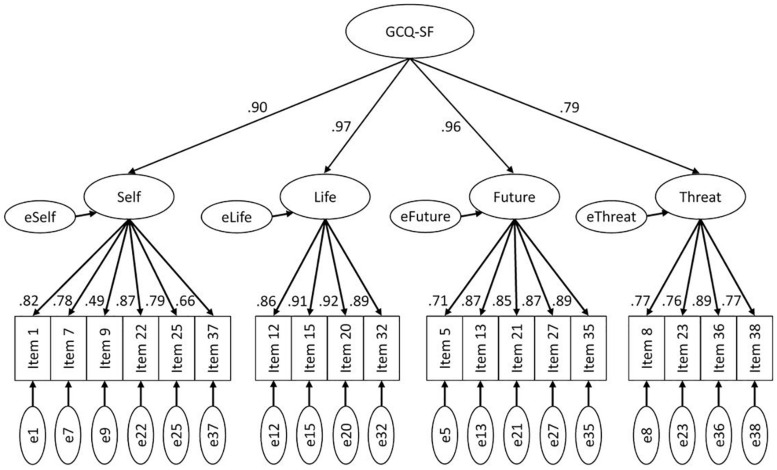
Confirmatory factor analysis of the GCQ-SF (model 1-SF). Path diagram for the confirmatory factor analysis of the GCQ-SF with four intercorrelated factors representing the four included subscales and a general factor. Error terms are denoted with a small ‘e’. All path coefficients are significant at *p* < 0.001.

Confirmatory factor analyses examined the factorial structure of the GCQ and the GCQ-SF. Table 2 presents the fit indices for the respective models. For the original GCQ, model 1 (nine correlated factors) demonstrated a better fit to the data on all absolute fit indices and especially on the comparative indices (CFI, AIC) than model 2 (second-order nine-factor model). For the GCQ-SF, model 1-SF stipulated four correlated factors representing the four included subscales. Model 2-SF added to this a general factor (second-order four-factor model). The goodness of fit indices are presented in Table 2. Model 1-SF demonstrated superior fit to model 2-SF according to all indices. Figure 1 illustrates the path diagram for model 1-SF; all regression weights were significant (*p* < 0.001). Please refer to the Supplementary Material for path diagrams for the model 2-SF (Supplementary Material 2) and the models 1 (Supplementary Material 3) and 2 (Supplementary Material 4) for the GCQ.

**TABLE 2:** Fit indices for the models tested in the confirmatory factor analyses.

Model	*χ*^2^	*df*	*p*	*χ*^2^/*df*	RMSEA	SRMR	CFI	TLI	AIC	BIC
1 (GCQ)	1980.50	629	<0.001	3.15	0.061	0.056	0.919	0.910	2204.50	2694.12
2 (GCQ)	2335.51	656	<0.001	3.56	0.066	0.069	0.900	0.892	2505.51	2877.10
1 SF (GCQ-SF)	709.44	146	<0.001	4.86	0.081	0.040	0.942	0.932	797.44	989.79
2 SF (GCQ-SF)	782.36	148	<0.001	5.29	0.086	0.048	0.935	0.925	866.36	1049.97

*Model 1 (GCQ): intercorrelated nine-factor model; model 2 (GCQ): nine factors with a higher order factor; model 1-SF (GCQ-SF): intercorrelated four-factor model for the abbreviated GCQ-SF; model 2-SF (GCQ-SF): four factors with a higher order factor for the abbreviated GCQ-SF. AIC, Akaike Information Criterion; BIC, Bayesian Information Criterion; CFI, Comparative Fit Index; TLI, Tucker-Lewis Index; RMSEA, Root Mean Square Error of Approximation; SRMR, Standardized Root Mean Residual.*

### Exploratory Analysis of Sociodemographic Variables

Age was not significantly associated with the GCQ (*r* = 0.072, *p* = 0.081). It demonstrated a significant but small association with the GCQ-SF (*r* = 0.157, *p* < 0.001): while the subscales ‘Self’ and ‘Threatening Interpretations of Grief’ showed no correlation with age, higher age was significantly associated with more endorsement of negative cognitions regarding ‘Life’ (*r* = 0.23, *p* < 0.001) and ‘Future’ (*r* = 0.24, *p* < 0.001). Women reported higher GCQ scores (53.59 ± 39.41) than men (32.87 ± 29.36). This difference was significant (*t*(99.72) = 5.20, *p* < 0.001, *d* = 0.60). The same difference was evident for the GCQ-SF (*t*(104.15) = 5.36, *p* < 0.001, *d* = 0.60) with women reporting higher scores (25.33 ± 23.24) than men (13.32 ± 16.35).

### Validity

Table 3 presents the zero-order correlations between the GCQ and grief severity (ICG-D), grief rumination (UGRS-D), optimism and pessimism (LOT-R) and symptoms of anxiety (HADS-D_anx_) and depression (HADS-D_depr_). Supporting convergent and discriminant validity, the GCQ-scores were positively associated with grief rumination and pessimism, and negatively with optimism. The same correlational pattern was evident for the GCQ-SF. Concerning criterion validity, the GCQ-scores were positively associated with grief severity. Fisher’s *z*-test demonstrated that the correlation of the GCQ with grief severity was higher than with depression (*z* = 5.767, *p* < 0.001) and anxiety (*z* = 8.254, *p* < 0.001). The same results were obtained for the respective correlations of the GCQ-SF with grief severity and depression (*z* = 2.811, *p* < 0.002) and grief severity and anxiety (*z* = 8.713, *p* < 0.001). These significant differences demonstrate a closer association between GCQ and GCQ-SF and grief severity than with other measures of psychopathology.

**TABLE 3:** Correlations of the GCQ, the GCQ-SF and the subscales with measures of optimism, pessimism, grief severity, grief rumination, depression and anxiety.

	LOT-pess	LOT- opt	ICG-D	UGRS-D	HADS-D Depression	HADS-D Anxiety
GCQ Sum Score	0.57***	−0.58***	0.82***	0.73***	0.72***	0.67***
GCQ-SF Sum Score	0.53***	−0.61***	0.81***	0.67***	0.76***	0.63***
Self†	0.54***	−53***	0.72***	0.63***	0.63***	0.57***
World	0.46***	−0.47***	0.68***	0.62***	0.57***	0.51***
Life†	0.44***	−0.54***	0.71***	0.56***	0.72***	0.52***
Future†	0.54***	−0.63***	0.76***	0.64***	0.80***	0.59***
Self-blame	0.35***	−0.34***	0.48***	0.53***	0.34***	0.41***
Others	0.39***	−0.39***	0.56***	0.58***	0.50***	0.48***
Appropriateness of Grief	0.34***	−0.29***	0.40***	0.37***	0.33***	0.42***
Cherish Grief	0.38***	−0.32***	0.55***	0.49***	0.42***	0.39***
Threatening Interpretations of Grief†	0.45***	−0.46***	0.70***	0.58***	0.58***	0.58***

***p < 0.001; Bonferroni corrected threshold: p < 0.0008 (all p with *** are significant after Bonferroni correction). Scales marked with † are included in the GCQ short form (GCQ-SF). GCQ, Grief Cognitions Questionnaire; HADS-D, Hospital Anxiety and Depression Scale, German version; ICG-D, Inventory of Complicated Grief, German version; LOT-pess, pessimism subscale of the Life Orientation Test-Revised; Lot-opt, optimism subscale of the Life Orientation Test-Revised; UGRS-D, Utrecht Grief Rumination Scale, German version.

### Comparison of the Criterion Validity of the GCQ and the GCQ-SF

The criterion probable prolonged grief ‘caseness’ (ICG ≥ 25 and time since loss ≥ 6 months) classified *n* = 238 participants as cases and *n* = 234 as non-cases. In the logistic regression, model 1 demonstrated a significant association between GCQ-SF scores and probable ‘caseness’ (χ^2^(4) = 293.77, *p* < 0.001; Nagelkerkes R^2^ = 0.63). All four GCQ-SF scales were significant predictors: ‘Self’ (Wald(1) = 6.58, *p* = 0.010; OR = 1.2, 95% CI, 1.02–1.22), ‘Life’(Wald(1) = 4.12, *p* = 0.042; OR = 1.18, 95% CI, 1.01–1.37), ‘Future’ (Wald(1) = 9.07, *p* = 0.003; OR = 1.14, 95% CI, 1.05–1.24), and ‘Threatening Interpretations of Grief‘ (Wald(1) = 10.62, *p* = 0.001; OR = 1.14, 95% CI, 1.05–1.24). With increasing GCQ-SF scores, the relative probability of being a member of the high-risk group for prolonged grief increased. Model 2 was also significant, selecting five of the nine subscales as predictors (χ^2^(5) = 303.28, *p* < 0.001). Table 4 presents the statistical tests for the individual predictors included in the final model. While all GCQ-SF subscales were included as predictors, of the remaining five GCQ subscales, only ‘Others’ contributed significantly to the prediction of group membership. The amount of variance explained by the second model was 64% (Nagelkerkes R^2^).

**TABLE 4:** Logistic regression of membership in high-risk vs low to medium risk for prolonged grief group on all Grief Cognitions Questionnaire (GCQ) subscales (model 2).

Predictor	β	SE β	Wald’s χ^2^	*df*	*p*	OR (95% CI)
Constant	–2.56	0.27	92.86	1	<0.001	0.07
Self†	0.08	0.05	3.28	1	0.070	1.09 (0.99–1.18)
Life†	0.19	0.08	5.40	1	0.020	1.21 (1.03–1.41)
Future†	0.10	0.05	4.59	1	0.032	1.10 (1.01–1.21)
Threatening Interpretations of Grief†	0.13	0.04	10.52	1	0.001	1.14 (1.05–1.23)
Others	0.11	0.04	9.29	1	0.002	1.12 (1.04–1.20)

*Scales marked with † are included in the GCQ short form (GCQ-SF). Method of entry: forward selection (Wald).*

**FIGURE 2:**
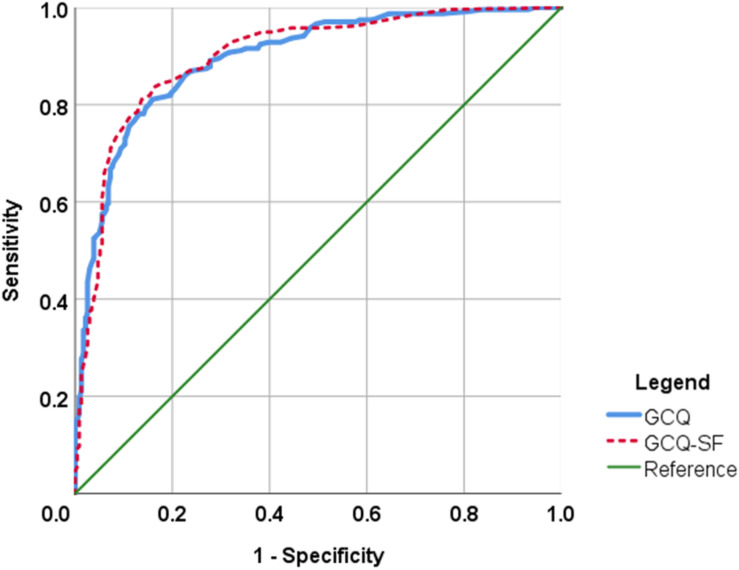
ROC curves of the GCQ and GCQ-SF. Comparison of sensitivity and specificity of the GCQ and GCQ-SF in predicting probable prolonged grief caseness (ICG ≥ 25 and time since loss ≥6 months).

Second, we conducted an ROC analysis to examine the sensitivity and specificity with which the GCQ and the GCQ-SF predicted probable ‘caseness’. For the GCQ, the analysis showed an AUC = 0.896 (SE = 0.014; *p* < 0.001; CI: 0.868–0.924); the GCQ-SF demonstrated an AUC = 0.900 (SE = 0.015; *p* < 0.001; CI: 0.871–0.928). See Figure 2 for a comparison of the sensitivity and specificity profiles.

## Discussion

This is the first study to investigate the psychometric properties of the frequently used abbreviated version (GCQ-SF) of the GCQ and to validate both the long and abbreviated versions of the GCQ in German. The German GCQ replicated the factor structure of the original GCQ, while for the abbreviated GCQ-SF, a four-factor structure with interrelated factors was proposed, representing its four subscales. Both questionnaires demonstrated very good item properties, excellent internal consistency and validity. Comparative analyses of the GCQ and GCQ-SF supported the usefulness of the abbreviated version: the GCQ-SF’s criterion validity is nearly identical to the GCQ, while using only four of the original nine subscales, including 19 of the original 38 items.

The GCQ and the GCQ-SF demonstrated excellent internal consistency. Internal consistencies of the subscales were good to excellent with the exception of ‘Cherish Grief’ (still satisfactory with Cronbach’s α = 0.76), which is part of the original GCQ but not the GCQ-SF. Item-whole correlations were medium to high showing slightly better psychometric properties for the GCQ-SF. The item difficulties were mostly in the medium range, which is desirable to achieve maximum discriminatory power. Five items (9, 11, 31, 34, 38) proved of high difficulty in our sample, i.e., were seldomly endorsed; only two of them are also part of GCQ-SF. Their difficulties suggest that many bereaved persons in our sample felt that these items did not describe their personal situation adequately. When interpreting this fact it is important to consider that negative cognitions after bereavement are, on the one hand, a very common finding in bereaved samples (Parkes, 1988; Schwartzberg and Janoff-Bulman, 1991; Rando, 1993), but on the other hand, a correlate of prolonged grief as a pathological grief reaction (Boelen et al., 2006b). It may thus make sense that some items may be relevant to many ‘healthy’ grievers, while others only apply to a minority of bereaved persons with more disabling grief reactions: hence, some items are endorsed less often. The more difficult items may be applicable to non-normative samples of bereaved persons and have discriminatory power among them, e.g., in treatment-seeking populations with higher bereavement-related distress or samples with special loss characteristics. To illustrate, item nine (‘I am ashamed of myself, since he/she died’) may only be relevant under certain circumstances, e.g., after bereavement by suicide, which is often associated with an increased perception of stigma (Hanschmidt et al., 2016). Future research investigating the psychometric properties of the GCQ could further clarify the utility of these items in different populations of bereaved persons, i.e., in samples of persons with clinically relevant bereavement-related distress and different loss characteristics.

The confirmatory factor analyses for the German GCQ mirrored the results for the original GCQ (Boelen and Lensvelt-Mulders, 2005). For the GCQ, we tested the two models identified as the best fitting in the original and found adequate fit indices indicating that the translated German version functions very similar to the original scale. While the model with nine interrelated factors demonstrated a better fit to the data on both the absolute fit indices and the comparative indices than the model including an additional higher-order factor, the fit indices of both models were in the same range. Thus, while acknowledging the distinctness of the subscales, the use of the total GCQ score as a general index of negative cognitions after bereavement seems still justified. For the GCQ-SF, the overall fit indices were below those of the long version. Nevertheless, the confirmatory factor analysis provided structurally very similar results, indicating a better fit for a model with four interrelated factors while still legitimizing the usage of the sum score of the scale. In addition to the fit indices, parsimony should also be considered when evaluating model fit, which would favor the higher-order models. On a content-level, the GCQ subscales are indicators of different themes of negative cognitions after bereavement. Depending on the respondents’ individual bereavement experience, some themes may be more relevant to their personal situation than others and not all will necessarily be endorsed similarly. This may explain the slightly better fit of lower-order models in our data for both GCQ and GCQ-SF. For research purposes and from the conceptual viewpoint of the cognitive behavioral model of prolonged grief (Boelen et al., 2006b), however, it seems essential to assess the overall role that negative cognitions plays in the bereavement response. The use of the sum score serves this function and can thus meaningfully contribute to grief research.

In an exploratory analysis, we investigated the association of sociodemographic variables with the GCQ and GCQ-SF. Age did not correlate significantly with the GCQ. Its association with the GCQ-SF, however, was significant and could be traced to two subscales. Older participants reported higher scores on the subscales ‘Life’ and ‘Future’, indicating more negative cognitions with regard to a life and future without the deceased. This could reflect the increasing difficulty of envisioning, and adapting to, a life after the loss for older bereaved individuals. We also found an effect of gender on the GCQ and the GCQ-SF, with women reporting higher scores than men. Women may be more likely to endorse or to report negative grief-specific cognitions. On the other hand, this finding must be interpreted with caution, because our sample was predominantly female and our analysis thus cannot control for confounding factors such as age, relationship to the deceased and other loss-related variables. While our results thus highlight the need to investigate gender differences in grief-specific cognitions, a detailed analysis should be reserved for more gender-balanced samples that allow controlling loss-related factors.

With regard to validity, our results align well with, and corroborate, previous findings from cross-sectional and prospective studies (Boelen et al., 2003, 2006a; Boelen and Lensvelt-Mulders, 2005; Boelen and Klugkist, 2011). As expected, both versions of the GCQ correlated positively with pessimism and negatively with optimism. Both GCQ versions also demonstrated substantial associations with symptoms of prolonged grief, depression and anxiety. Importantly, however, their associations with prolonged grief symptoms were significantly stronger than those with indicators of other types of psychopathology, thus strengthening the argument of a specific association of the GCQ (and the GCQ-SF) with these symptoms. While a correlation in the medium range has been reported between the GCQ and a three-item *ad-hoc* scale of grief-related rumination (Boelen and Lensvelt-Mulders, 2005), our study found a high correlation between the GCQ (and the GCQ-SF) and a validated scale of grief-specific rumination (UGRS-D). This closer association between thought content (GCQ/GCQ-SF) and ruminative thought processes (UGRS-D) is probably explained by the use of different measures in previous research and our study.

Since these results speak for the good psychometric properties of both GCQ versions, we were additionally interested in a comparison of both questionnaires. To compare the GCQ and the GCQ-SF with regard to their criterion validity, we first established probable prolonged grief ‘caseness’ in our sample based on the cut-off score in the ICG (Kristensen et al., 2010; Newson et al., 2011) and the ICD-11 time criterion (World Health Organization [WHO], 2020). In the logistic regression to predict group membership, the forward selection method included all four GCQ-SF subscales in the models, while only one of the remaining five GCQ subscales was a significant predictor (‘Others’). This second model including ‘Others’, however, contributed only one additional percent of explained variance to the variance already explained by the four GCQ-SF subscales. In this analysis, the GCQ-SF thus performs nearly identical to the GCQ, which is twice as long. In a second analysis, we used a ROC approach to investigate the questionnaires’ discriminatory power, also using probable ‘caseness’ of prolonged grief as criterion. Again, the GCQ-SF performed equally well as the GCQ, with a negligible difference in AUCs of 0.004 favoring the GCQ-SF. This means that both questionnaires share the same properties in relative specificity and sensitivity.

However, it is important to consider that the GCQ-SF provides only information about the grief-specific cognitions concerning ‘Self’, ‘Life’, ‘Future’ and ‘Threatening Interpretations of Grief’. Whenever a more comprehensive assessment of negative beliefs is desirable, especially for clinical purposes, the original GCQ with its five additional subscales may be better suited to the task. In our study, the additional subscales of the original GCQ (‘World’, ‘Self-Blame’, ‘Others’, ‘Appropriateness’, ‘Cherish Grief’) added only little to its criterion validity (i.e., explanation of variance in probable prolonged grief ‘caseness’). This suggests that these five themes are less important to prolonged grief (as specified in our provisional diagnostic category). However, this conclusion is based on a group-level analysis of a non-treatment-seeking sample with varying time since loss. It does not preclude that in individual cases these five themes may be of high relevance to individual grief distress, e.g., self-blame following the death of a spouse (Field and Bonanno, 2001). These considerations point to the fact that the GCQ and GCQ-SF may have differential fields of application. Importantly, however, the results of our study clearly show that the GCQ-SF, in spite of its brevity, preserves all positive psychometric properties of the original questionnaire.

### Strengths and Limitations

Strengths of our study are the large sample size, the careful backtranslation procedure in developing the German GCQ in cooperation with one of the authors of the original GCQ, and the use of well-validated questionnaires in the assessment of all constructs. Our recruitment strategies allowed us to reach out to, and include, a wide age-range of participants with different bereavement experiences, a wide range of time since loss and varying levels of grief-related distress. In our analysis of discriminatory power of the GCQ, we considered the cut-off for ‘caseness’ of prolonged grief established in the research tradition of the field and combined it with the most recent time-criterion for PGD, thus facilitating the interpretation of our results within a dynamic and changing classification of pathological grief reactions.

At the same time, some limitations of our study must be taken into account. First, our design is cross-sectional and does therefore not allow for causal interpretation. Second, our sample was predominantly female (88%), thus the generalizability of our findings remains to be tested. While the use of convenience sampling and some of our various recruitment strategies may have contributed to the present sampling bias, an overrepresentation of female participants in bereavement research has been reported as a general methodological problem in this field of research previously (Stroebe et al., 2003). Future research should try to implement recruiting strategies that oversample hitherto underrepresented populations, especially bereaved men. Samples that are more balanced would also allow for detailed analyses of the preliminary gender effect, which was evident in our data. Third, all our measures were based on self-report. Self-report is an adequate way of assessing cognitions, i.e., private mental processes. However, for clinical and diagnostic purposes, i.e., when establishing the possible caseness for prolonged grief, a more thorough assessment than the ICG, such as a clinical interview, is desirable. This is especially important when considering the findings of our discriminatory power analyses where we established a tentative caseness for prolonged grief based on the German version of the self-report measure ICG. The ICG is a very well-validated instrument for assessing grief-related distress: nevertheless, it does not assess all symptoms of PGD and PCBD (for a discussion: (Eisma et al., 2020)), and no clinical diagnosis can be made based on self-report only. Fourth, our GCQ-SF results are based on data of participants who answered the complete GCQ. While we found no evidence for negative effects of the serial position of the items in the survey (Galesic and Bosnjak, 2009), we cannot exclude priming effects due to the presentation of a wider range of negative cognitions. Lastly, although our confirmatory factor analyses yielded adequate results, which were comparable to findings on the original GCQ, the fit indices reflect an adequate rather than an excellent fit; this is particularly true with regard to the short version and indicates that the model specification could still be optimized.

### Future Research and Implications

The present study validated a German version of the GCQ and additionally investigated the psychometric properties of its frequently used, abbreviated form GCQ-SF. While both questionnaires demonstrated good to excellent psychometric properties, our study also suggests different areas of future research and applicability for both questionnaires.

The original GCQ’s strength lies within its comprehensive assessment of various themes of negative cognitions after bereavement in the clinical setting, i.e., the provision of individual grief counseling and therapy. It offers the health care provider a chance to assess all relevant cognitive themes comprehensively and at the same time more efficiently than in an interview. Themes that are of high individual relevance to the client can then be followed-up by a more in-depth exploration. The GCQ can thus help to identify a client’s dysfunctional cognitions after bereavement and provide a starting point for psychotherapeutic interventions like cognitive restructuring, which is an efficient and widely employed strategy in PGD treatment (Doering and Eisma, 2016). The GCQ can also serve as a tool to assess treatment progress, i.e., by comparing scores before and after treatment. The change sensitivity of the GCQ-SF’s total score after cognitive behavioral therapy has already been shown in psychotherapy research (Boelen et al., 2011). Future research should investigate whether changes in individual items of the original GCQ delineate the effects of cognitive restructuring on specific, personally highly relevant themes in therapy, thus informing health care providers’ focus on an individual patient level. In this way, future research could address the GCQ’s ability to contribute to planning and evaluation of therapy. Through its comprehensiveness, the GCQ also offers another very interesting line for future research: It could serve to identify cognitive themes that are of special relevance to distinct bereavement experiences. By recruiting a treatment-seeking sample with varying bereavement-related characteristics (e.g., type of loss, causes of death, expectedness vs unexpectedness of death) and administering the GCQ, research could systematically investigate the associations between these characteristics and specific cognitive themes they may give rise to, thus providing empirical evidence to support the development of more population-tailored interventions.

In contrast, the GCQ-SF is limited to 19 items, which capture cognitions that are more widely endorsed (i.e., it contains fewer items with high item difficulty). Its brevity saves time compared to the original GCQ. While this aspect may not be of special relevance in individual therapy, it may offer important advantages in other settings. Efficacy or effectiveness research of grief therapy needs reliable, change-sensitive and relatively short outcome measures (Rosner, 2015). This is especially important since study participants often face many time points of assessment, all including a whole package of questionnaires. In this context, 19 additional items may make a difference to the patients’ commitment to answer the questionnaires conscientiously and to their burden of grief-related distress in the diagnostic assessment (Galesic and Bosnjak, 2009). Since we still know too little about the relative effectiveness of different treatment components in PGD treatment (Doering and Eisma, 2016), being able to assess treatment effects on changes in cognitions effectively and efficiently could contribute to future research and a better understanding of what works in grief therapy. The GCQ-SF seems very well suited to this task since – in spite of its brevity – it preserves all positive psychometric properties of the original GCQ.

In conclusion, both the GCQ and the GCQ-SF have the potential to contribute to a better understanding of negative cognitions after bereavement and to the improvement of health care provision. Establishing validated versions of these questionnaires and making them available to the interested public serves this wider goal.

## Data Availability Statement

The raw data supporting the conclusions of this article will be made available by the authors, without undue reservation.

## Ethics Statement

The study involving human participants was reviewed and approved by Ethics Committee of the Department of Psychology from the Philipps-University Marburg (Germany). The patients/participants provided their written informed consent to participate in this study.

## Author Contributions

BD and PB conceptualized the project. BD and AB jointly administrated the project and data curation, conducted the data analysis and interpretation, and drafted the manuscript. ME and PB contributed to data interpretation and editing the manuscript. All authors reviewed the manuscript and provided approval for the final version.

## Conflict of Interest

The authors declare that the research was conducted in the absence of any commercial or financial relationships that could be construed as a potential conflict of interest.
