# Supplementary material for: Validation of a German Version of the Grief Cognitions Questionnaire and Establishment of a Short Form
Source: Front Psychol. 2021 Jan 18;11:620987. doi: 10.3389/fpsyg.2020.620987 (PMC7848142; doi:10.3389/fpsyg.2020.620987)
Supplement: Supplementary file 1 [file Presentation_1.PPTX]

## Slide 1
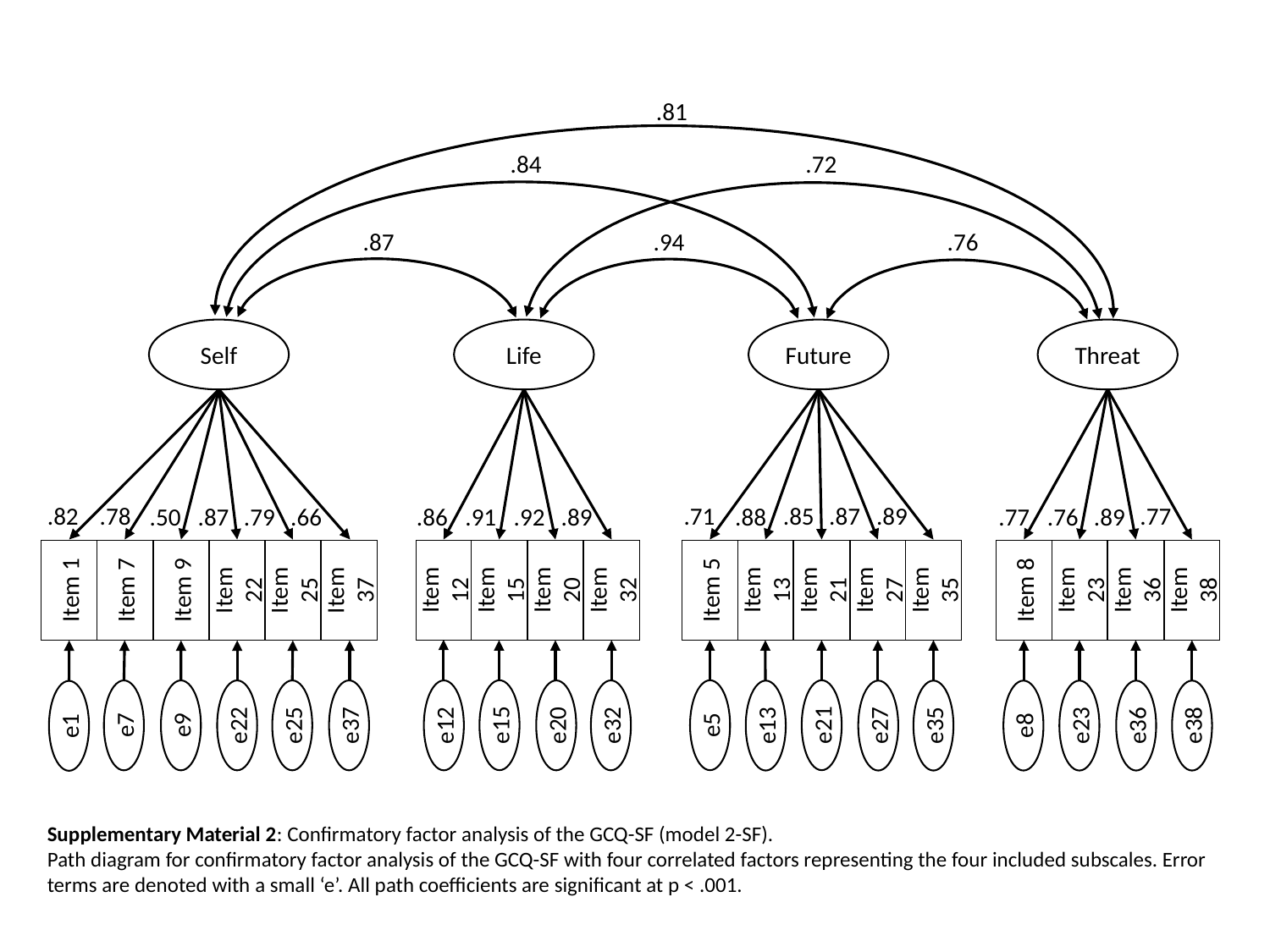

.81
.84
.72
.94
.76
.87
Self
Life
Future
Threat
.77
.71
.78
.82
.87
.89
.85
.50
.88
.79
.87
.89
.77
.76
.66
.89
.92
.91
.86
Item 12
Item 15
Item 20
Item 32
Item 5
Item 13
Item 21
Item 27
Item 35
Item 8
Item 23
Item 36
Item 38
Item 1
Item 7
Item 9
Item 22
Item 25
Item 37
e15
e5
e21
e32
e20
e7
e9
e22
e25
e37
e12
e38
e36
e23
e8
e13
e27
e35
e1
Supplementary Material 2: Confirmatory factor analysis of the GCQ-SF (model 2-SF).
Path diagram for confirmatory factor analysis of the GCQ-SF with four correlated factors representing the four included subscales. Error terms are denoted with a small ‘e’. All path coefficients are significant at p < .001.
