# Supplementary material for: Validation of a German Version of the Grief Cognitions Questionnaire and Establishment of a Short Form
Source: Front Psychol. 2021 Jan 18;11:620987. doi: 10.3389/fpsyg.2020.620987 (PMC7848142; doi:10.3389/fpsyg.2020.620987)
Supplement: Supplementary file 3 [file Presentation_3.PPTX]

## Slide 1
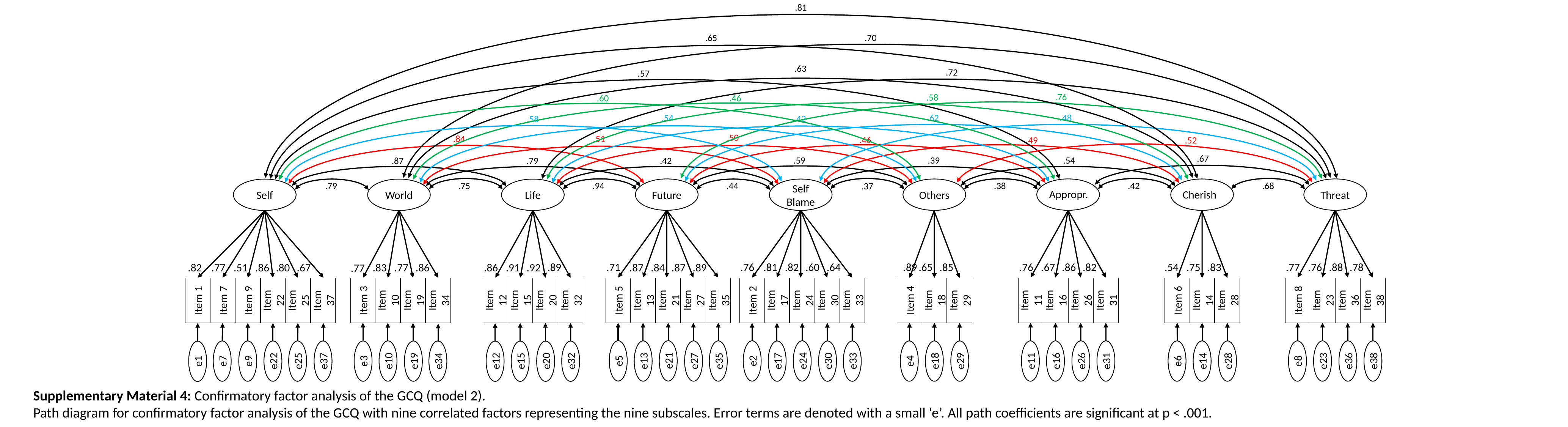

.81
.65
.70
.63
.72
.57
.76
.58
.46
.60
.62
.48
.54
.42
.58
.50
.84
.51
.46
.49
.52
.67
.59
.54
.39
.42
.79
.87
.94
.79
.75
.38
.44
.42
.68
.37
Future
Threat
Others
Self Blame
Self
World
Life
Appropr.
Cherish
.86
.88
.76
.67
.76
.83
.54
.85
.89
.75
.65
.76
.60
.64
.82
.77
.81
.82
.78
.89
.71
.77
.82
.87
.89
.84
.87
.51
.80
.86
.92
.86
.91
.67
.86
.77
.83
.77
Item 5
Item 13
Item 21
Item 27
Item 35
Item 11
Item 16
Item 26
Item 31
Item 8
Item 23
Item 36
Item 38
Item 6
Item 14
Item 28
Item 4
Item 18
Item 29
Item 2
Item 17
Item 24
Item 30
Item 33
Item 3
Item 10
Item 19
Item 34
Item 12
Item 15
Item 20
Item 32
Item 1
Item 7
Item 9
Item 22
Item 25
Item 37
e5
e21
e2
e24
e31
e38
e26
e36
e16
e23
e11
e8
e13
e27
e35
e10
e28
e14
e6
e29
e18
e4
e34
e19
e7
e9
e22
e25
e37
e3
e17
e30
e33
e32
e20
e15
e12
e1
Supplementary Material 4: Confirmatory factor analysis of the GCQ (model 2).
Path diagram for confirmatory factor analysis of the GCQ with nine correlated factors representing the nine subscales. Error terms are denoted with a small ‘e’. All path coefficients are significant at p < .001.
