# Supplementary material for: Validation of a German Version of the Grief Cognitions Questionnaire and Establishment of a Short Form
Source: Front Psychol. 2021 Jan 18;11:620987. doi: 10.3389/fpsyg.2020.620987 (PMC7848142; doi:10.3389/fpsyg.2020.620987)
Supplement: Supplementary file 4 [file Table_1.DOCX]

Supplementary Material 1:

Grief Cognitions Questionnaire (German Version)

Im Folgenden finden Sie eine Reihe negativer Überzeugungen. Bitte geben Sie an, wie sehr Sie jeder dieser Überzeugungen zustimmen.

|  | Stimme über-haupt nicht zu |  |  |  |  | Stimme voll-kommen zu |
| --- | --- | --- | --- | --- | --- | --- |
| 1. Seit er/sie tot ist, denke ich, dass ich wertlos bin. | ⓪ | ① | ② | ③ | ④ | ⑤ |
| 2. Ich bin mitverantwortlich für seinen/ihren Tod. | ⓪ | ① | ② | ③ | ④ | ⑤ |
| 3. Seit er/sie gestorben ist wird mir klar, dass die Welt ein schlechter Ort ist. | ⓪ | ① | ② | ③ | ④ | ⑤ |
| 4. Die Menschen in meinem Umfeld sollten mich mehr unterstützen. | ⓪ | ① | ② | ③ | ④ | ⑤ |
| 5. Ich erwarte nicht, dass es mir in der Zukunft besser gehen wird. | ⓪ | ① | ② | ③ | ④ | ⑤ |
| 6. Ich muss trauern, ansonsten werde ich vergessen. | ⓪ | ① | ② | ③ | ④ | ⑤ |
| 7. Ich sehe mich als einen schwachen Menschen, seit er/sie verstorben ist. | ⓪ | ① | ② | ③ | ④ | ⑤ |
| 8. Wenn ich meinen Gefühlen freien Lauf lasse, werde ich verrückt. | ⓪ | ① | ② | ③ | ④ | ⑤ |
| 9. Ich schäme mich, seit er/sie gestorben ist. | ⓪ | ① | ② | ③ | ④ | ⑤ |
| 10. Sein/ihr Tod hat mir gezeigt, dass wir in einer furchtbaren Welt leben | ⓪ | ① | ② | ③ | ④ | ⑤ |
| 11. Meine Trauerreaktionen sind unnormal. | ⓪ | ① | ② | ③ | ④ | ⑤ |

|  | Stimme über-haupt nicht zu |  |  |  |  | Stimme voll-kommen zu |
| --- | --- | --- | --- | --- | --- | --- |
| 12. Das Leben hat mir nichts mehr zu bieten. | ⓪ | ① | ② | ③ | ④ | ⑤ |
| 13. Ich habe keine Zuversicht in die Zukunft | ⓪ | ① | ② | ③ | ④ | ⑤ |
| 14. So lange ich trauere, erhalte ich die Verbundenheit zu ihm/ihr aufrecht. | ⓪ | ① | ② | ③ | ④ | ⑤ |
| 15. Mein Leben ist nutzlos seit seinem/ihrem Tod. | ⓪ | ① | ② | ③ | ④ | ⑤ |
| 16. Ich trauere nicht so, wie ich es sollte. | ⓪ | ① | ② | ③ | ④ | ⑤ |
| 17. Ich hätte seinen/ihren Tod verhindern sollen. | ⓪ | ① | ② | ③ | ④ | ⑤ |
| 18. Viele Menschen haben mich seit dem Tod von ihm/ihr enttäuscht. | ⓪ | ① | ② | ③ | ④ | ⑤ |
| 19. Sein/ihr Tod hat mich gelehrt, dass die Welt ungerecht ist. | ⓪ | ① | ② | ③ | ④ | ⑤ |
| 20. Mein Leben ist bedeutungslos, seit er/sie gestorben ist. | ⓪ | ① | ② | ③ | ④ | ⑤ |
| 21. Meine Wünsche für die Zukunft werden sich nie erfüllen. | ⓪ | ① | ② | ③ | ④ | ⑤ |
| 22. Seit er/sie tot ist, fühle ich mich weniger wertvoll. | ⓪ | ① | ② | ③ | ④ | ⑤ |
| 23. Wenn ich vollständig realisieren würde, was sein/ihr Tod bedeutet, würde ich verrückt werden. | ⓪ | ① | ② | ③ | ④ | ⑤ |
| 24. Wenn ich etwas anders gemacht hätte, wäre er/sie noch am Leben. | ⓪ | ① | ② | ③ | ④ | ⑤ |

|  | Stimme über-haupt nicht zu |  |  |  |  | Stimme voll-kommen zu |
| --- | --- | --- | --- | --- | --- | --- |
| 25. Seitdem er/sie gestorben ist, denke ich negativ von mir selbst. | ⓪ | ① | ② | ③ | ④ | ⑤ |
| 26. Ich reagiere auf diesen Verlust nicht normal. | ⓪ | ① | ② | ③ | ④ | ⑤ |
| 27. In der Zukunft werde ich nie wieder wirklich glücklich werden. | ⓪ | ① | ② | ③ | ④ | ⑤ |
| 28. So lange ich trauere, muss ich ihn/sie nicht wirklich gehen lassen. | ⓪ | ① | ② | ③ | ④ | ⑤ |
| 29. Die Menschen in meinem Umfeld sollten viel mehr auf mich eingehen. | ⓪ | ① | ② | ③ | ④ | ⑤ |
| 30. Ich werde mir nie für die Dinge, die ich in der Beziehung zu ihm/ihr falsch gemacht habe, vergeben können. | ⓪ | ① | ② | ③ | ④ | ⑤ |
| 31. Mit meinen Gefühlen stimmt etwas nicht. | ⓪ | ① | ② | ③ | ④ | ⑤ |
| 32. Mein Leben hat keinen Sinn mehr, seit er/sie gestorben ist. | ⓪ | ① | ② | ③ | ④ | ⑤ |
| 33. Ich mache mir Vorwürfe, dass ich mich nicht besser um ihn/sie gekümmert habe. | ⓪ | ① | ② | ③ | ④ | ⑤ |
| 34. Sein/ihr Tod von hat mich gelehrt, dass die Welt ein wertloser Ort ist. | ⓪ | ① | ② | ③ | ④ | ⑤ |
| 35. Seit er/sie nicht mehr da ist, habe ich eine negative Perspektive auf die Zukunft. | ⓪ | ① | ② | ③ | ④ | ⑤ |
| 36. Wenn ich meine Gefühle aufkommen lasse, werde ich die Kontrolle verlieren. | ⓪ | ① | ② | ③ | ④ | ⑤ |

|  | Stimme über-haupt nicht zu |  |  |  |  | Stimme voll-kommen zu |
| --- | --- | --- | --- | --- | --- | --- |
| 37. Seit er/sie tot ist, bin ich niemandem mehr wichtig. | ⓪ | ① | ② | ③ | ④ | ⑤ |
| 38. Wenn ich einmal anfangen würde zu weinen, würde ich die Kontrolle verlieren | ⓪ | ① | ② | ③ | ④ | ⑤ |

Der Gesamtwert des GCQ wird durch Summierung der Einzelitems gebildet. Subskalenwerte berechnen sich durch Summierung der zugehörigen Items:

1. Selbst: Items 1, 7, 9, 22, 25, 37
2. Welt: Items 3, 10, 19, 34
3. Leben: Items 12, 15, 20, 32
4. Zukunft: Items 5, 13, 21, 27, 35
5. Selbstvorwürfe: Items 2, 17, 24, 30, 33
6. Andere: Items 4, 18, 29
7. Angemessenheit: Items 11, 16, 26, 31
8. Festhalten: Items 6, 14, 28
9. Bedrohliche Interpretation von Trauer: Items 8, 23, 36, 38
